# Burnout chez les professionnels soignants de l'Hôpital Central de Yaoundé

**DOI:** 10.11604/pamj.2019.34.126.19969

**Published:** 2019-06-21

**Authors:** Annicet Bopda Negueu, Samuel Nambile Cumber, Layu Donatus, Claude Ngwayu Nkfusai, Bestina Forkwa Ewang, Fala Bede, Terrence Epie Beteck, Joyce Shirinde, Vincent de Paul Djientcheu, Benjamin Alexandre Nkoum

**Affiliations:** 1 Department of Public Health, School of Health Sciences, Catholic University of Central Africa, Yaoundé, Cameroon; 2 Faculty of Health Sciences, University of the Free State, Bloemfontein, South Africa; 3 Section for Epidemiology and Social Medicine, Department of Public Health, Institute of Medicine, The Sahlgrenska Academy at University of Gothenburg, Box 414, SE-405 Gothenburg, Sweden; 4 School of Health Systems and Public Health, Faculty of Health Sciences, University of Pretoria Private Bag X323, Gezina, Pretoria, South Africa; 5 Yaounde Central Hospital, Cameroon; 6 Cameroon Baptist Convention Health Services, Yaounde, Cameroon

**Keywords:** Burnout, facteurs associés, personnels soignants, Hôpital Central de Yaoundé, Burnout, associated factors, caregivers, healthcare workers (HCW), Yaounde Central Hospital

## Abstract

**Introduction:**

Le burnout ou syndrome d'épuisement professionnel des soignants est un problème de santé publique au Cameroun. Il se manifeste par un épuisement émotionnel, une dépersonnalisation et une diminution de l'accomplissement personnel du sujet. Il touche la plupart des personnels soignants et les conséquences sont nombreuses. Au Cameroun en général et à l'Hôpital Central de Yaoundé (HCY) en particulier, toutes ces dernières années, les soignants n'ont cessés d'exprimer leur mécontentement face à leurs conditions de travail à travers des grèves et menaces de divers ordres. La prise en charge des patients se fait de façon impersonnelle et on assiste à des conséquences dramatiques et des problèmes déontologiques.

**Méthodes:**

Notre étude transversale analytique avait pour objectif de déterminer les facteurs qui sont associés au burnout dans cette population des soignants de l'HCY. Pour ce faire, pendant un mois, nous avons administré auprès de ces soignants notre questionnaire conçu selon les modèles théoriques de Maslach et Siegrist. Nous avons pu obtenir des informations recherchées auprès de 104 personnels soignants; la saisie et l'analyse des données se sont faites avec SPSS 20.

**Résultats:**

Les résultats montrent auprès des soignants de quatre unités de soins de l'HCY des manifestations similaires à celles trouvées dans la littérature et une prévalence du Burnout (BO) de 63% parmi ces soignants. Sept facteurs y ont été associés de façon statistiquement significative: il s'agit du pavillon d'exercice (OR= 3.93; 1.16-13.24; p-value=0.027); le statut matrimonial (OR: 2.56; 1.22 - 5.39; p-value=0,049); le ratio effort/récompenses déséquilibré (OR: 2.31; 1.10 - 4.84; p-value=0.026 ); avoir été menacé physiquement ou verbalement (OR: 3,75; 1,49 - 9,41; 0,005); le maintien de l'équilibre entre vie privée et vie professionnelle (OR: 3,41; 1,19- 10,7; p-value=0,038); la fréquence des oublis (OR: 4,25 -1,33; 7,91; p-value=0,002) l'attribution des erreurs aux conditions de travail (OR: 2,05;1,52 - 24,0; p-value=0,011).

**Conclusion:**

Le burnout est présent au sein des populations soignantes de l'HCY et risque de s'accroitre si rien n'est fait. Des stratégies de prévention et de promotion de la santé au travail sont vivement nécessaires dans les aspects de l'amélioration des conditions de travail; les prises de bonnes décisions politique et managériales; l'amélioration des relations entre soignants et soignant-hiérarchie et la recherche constante, le suivi et contrôle des facteurs de risque.

## Abstract (en)

**Introduction:**

Burnout or burnout syndrome is a public health problem in Cameroon. It manifests itself by emotional exhaustion, depersonalization, and diminished personal accomplishment of the subject. It affects most healthcare workers (HCW) and the consequences are numerous. In Cameroon in general, and Yaounde Central Hospital (HCY) in particular, in recent years, healthcare workers have continued to express dissatisfaction with their working conditions through strikes and threats of various kinds. The care of patients is suboptimal with sometimes dramatic consequences and ethical concerns.

**Methods:**

Our cross-sectional study aimed to determine the factors that are associated with burnout among healthcare personnel (HCW) of HCY. To do this, for one month, we administered to these HCWs our questionnaire designed according to the theoretical models of MASLACH and SIEGRIST. We were able to obtain information from 104 caregivers; data entry and analysis was done with SPSS 20.

**Results:**

The results show that HCWs of four departments of HCY have similar manifestations to those found in the literature and the prevalence of burnout syndrome amon HCWs of HCY was 63%. Seven factors were statistically significantly associated with burnout syndrome among HCWs in HCY: the department (care unit) the HCW was assigned to (OR = 3.93, 1.16-13.24, p-value = 0.027); marital status (OR: 2.56, 1.22 - 5.39, p-value = 0.049); the effort-reward imbalance (OR: 2.31, 1.10 - 4.84, p-value = 0.026); having received been threatened physically or verbally (OR: 3.75, 1.49 - 9.41, 0.005); maintaining the balance between private and professional life (OR: 3.41, 1.19- 10.7, p-value = 0.038); frequency of forgetfulness (OR: 4.25 -1.33, 7.91, p-value = 0.002) and attribution of errors to working conditions (OR: 2.05, 1.52 - 24.0; -value = 0.011).

**Conclusion:**

Burnout is common among HCWs of HCY and is likely to keep increasing if nothing is done. Prevention and promotion strategies for occupational health are strongly needed in aspects of improving working conditions; making good political and managerial decisions; improving relationships between caregivers and caregivers-hierarchy and constant research, monitoring and control of risk factors.

## Introduction

L'épuisement professionnel, mieux connu sous le terme de « burnout », représente de nos jours un réel problème de santé publique avec des conséquences lourdes pour la santé des travailleurs, des entreprises et de la société en général [1]. À n'en croire qu'à la récurrence des épisodes médiatisés, à la tournure parfois dramatique (insultes, violences physiques, accidents de travail, grève et menaces de grève), la souffrance des soignants semble s'accroître au cours de ces dernières années [2]. C'est ainsi que le malaise de ces deniers est une réalité attestée par un certain nombre de recherches internationales qui rendent compte de l'importance du stress dans ce secteur professionnel [3]. On peut donc se rendre compte aujourd'hui que, soigner devient difficile, contraignant, voire très stressant [4]. En tant que métier de l'interaction, engageant la subjectivité à forte implication émotionnelle, il peut générer diverses tensions (céphalées, insomnies, irritabilité) et surtout aboutir à l'épuisement professionnel ou burnout caractérisé par un sentiment de fatigue extrême, de la dépression, un sentiment d'incapacité, un dégoût du travail, un désintérêt pour autrui [5]. L'épuisement professionnel nuit à l'individu tout autant qu'à l'organisation: il abaisse la performance et la satisfaction au travail, peut compromettre la qualité des soins, et accroît l'absentéisme et le turnover [6]. En France [2] ont rapporté à travers des études réalisées en 2007, des taux de Burnout de 46,5% et 30%. De même Shanafelt *et al.* ont démontré en 2012 que 65% des soignants américains avaient le Burnout et que 40,2% d'entre eux n'étaient plus satisfaits de leur travail [7]. En Australie [8] ont retrouvé en 2013 un taux de 60% de Burnout parmi les soignants des secteurs des urgences. En Afrique, toutefois, les études menées dans quelques pays comme le Sénégal, l'Egypte, le Maroc, avaient montrées une prévalence du burnout avoisinant 50%. Au Bénin, une étude réalisée à Cotonou en 2007 a montré que la prévalence du stress parmi les agents de santé était de 69,5% [9]. En Tunisie, une étude réalisée par Baddi, (2014) en milieu psychiatrique avait montré que 35,8% sur les médecins et infirmiers en Egypte, la plupart des participants (66%) avaient un niveau modéré de l´épuisement professionnel [9]. Au Cameroun par contre aucune étude concernant ce sujet n'a été publiée. Les professionnels de soins semblent subir des facteurs de stress plus important pouvant conduire à des taux élevés de Burnout. L'objectif de ce travail était de déterminer la fréquence du burnout et d'identifier les facteurs associés.

## Méthodes

Il s'agissait d'une étude transversale analytique, réalisée pendant le mois d'Octobre 2016 dans un hôpital public de référence de la ville de Yaoundé au Cameroun à savoir: l'Hôpital Central de Yaoundé.

**Echantillonage:** la population cible était constituée des personnels soignants des services des urgences médicales, urgences chirurgicales, de maternité et de réanimation/soins intensifs. Le choix de ces services a été basé sur le fait qu´ils sont des services ou les soins se font le plus souvent en urgence et par conséquent les personnels sont fréquemment exposés au stress. Ont été inclus dans l'étude, les personnels soignants des deux genres, exerçant dans les services sus cités de l'étude, présents au moment de l'étude, volontaires. Il s'agissait des médecins, infirmiers et aides-soignants. Les personnels exerçant exclusivement administratifs, ceux en congé ou de retour de congés et ceux ayant exercé il y'a moins de 6 mois ont été exclu. La formule de Lorentz a été utilisée pour calculer la taille minimale de l'échantillon avec la prévalence de 50% du burnout rapportée dans une étude chez le personnel soignant en Afrique. La taille minimale ainsi calculée était de 94 participants (avec un niveau de confiance a 95% et une marge d'erreur de 5%).

### Collecte des données

**L'évaluation du burnout a été réalisée par deux échelles:** les données ont été recueillies via l'administration d'une auto questionnaire anonyme distribué à chaque soignant. L'échelle MBI (Maslach Burnout Inventory) qui caractérise le burnout et permet d’explorer ses 3 dimensions: l'épuisement émotionnel (exploré par 9 items), la dépersonnalisation (explorée par 5 items) et l´accomplissement personnel (exploré par 8 items) a été utilisé pour établir la fréquence. Les modalités de réponses pour les 22 items sont basées sur une échelle de fréquence de 7 points avec une intensité allant de 0 à 6: « 0 = jamais » à « 6 = chaque jour ». Le score dans chaque dimension permet de déterminer le degré d'atteinte en élevé, modéré ou faible. Cette échelle est la plus utilisée dans la littérature pour décrire chaque dimension du burnout en milieu professionnel. Un auto-questionnaire anonyme, formé de 30 questions, a été élaboré pour le recueil: des caractéristiques sociodémographiques et professionnelles des sujets interrogés: 8 questions (âge, genre, situation matrimoniale, nombre d'enfants en charge); les conditions socioprofessionnelles (lieu d'exercice professionnel, ancienneté professionnelle, nombre d'heures quotidiennes de travail) et 22 questions pour le Maslach Burnout Inventory (MBI). L'échelle de Siegrist (33 questions) a permis de mesurer l'effort (6 questions), la récompense (11 questions), le surinvestissement (06 questions) et les facteurs associés (10 questions). Selon MBI il existe deux manières de catégoriser le patient en du burnout: soit dimension par dimension: ainsi on est atteint de burnout si l'Epuisement émotionnel (EE) est élevée, ou lorsque la Dépersonnalisation (DP) élevée ou lorsque l'Accomplissement personnel (AP) faible; soit en donnant un score global au sujet à partir de ces dimensions: burnout faible si une seule dimension est atteinte: EE élevé ou DP élevée ou AP bas, burnout modéré si deux dimensions sont atteintes: EE + DP, ou EE + AP, DP + AP, burnout sévère si les trois dimensions atteintes: EE élevé + DP élevée + AP bas. Le questionnaire était remis aux participants pour un délai d'au moins trois jours afin d'avoir suffisamment de temps pour s'en approprier.

**Traitement et analyse des données:** les données ont été entrées dans Microsoft Excel et analysées à l'aide de SPSS v20. Les fréquences et les pourcentages ont été déterminés pour les variables qualitatives. Nous avons utilisé le test du chi carré pour étudier l'association entre les caractéristiques sociodémographiques et la variable de résultat (présence ou non du syndrome de l'épuisement professionnel), tandis qu'une analyse de régression logistique univariée a été utilisée pour rechercher des associations à d'autres facteurs liés au syndrome d'épuisement professionnel identifiés par la revue de littérature et la variable de résultat. Notre niveau de signification a été fixé à une valeur p <0,05.

**Considération éthique:** cette étude a été autorisée par la Direction de l'hôpital Central de Yaoundé et a reçu la clairance du Comité d'Ethique Institutionnel de la Recherche pour la Santé Humaine (CEIRSH) qui a donné son accord en délivrant une clairance éthique (Réf. No 2017/0474/CEIRSH/ESS/MSI).

**Déroulement de la collecte:** muni d'une autorisation du directeur de la formation sanitaire, nous nous sommes introduit auprès des chefs de service; les questionnaires ont été distribués aux personnels soignants pendant l'exercice de leur activité et ce, en tenant compte du planning de travail dans leurs services. Après avoir expliqué clairement les objectifs de notre travail de recherche au participant, un consentement libre et éclairé a été déclaré de la part de ces derniers interrogés.

## Résultats

**Caractéristiques sociodémographiques et professionnelles:** le genre féminin était majoritaire avec 57,7 % de femmes. La moyenne d'âge étant de 38,06 ans (±9,99) avec un minimum de 24 ans et un maximum de 57 ans. La tranche d'âge la plus représentée était celle des soignants de 20 et 29 ans (31,7%) et nous avons noté que 65 soignants (62,5%) vivaient avec conjoint et enfant à charge. Plusieurs catégories professionnelles avaient été recensées: aides-soignants (23%), infirmiers (22%), médecins (10%). Leur répartition dans les différents services de soins a été précisée comme suit service d'urgences médicales (20,2%), urgences chirurgicales (15,4%) maternité (44,2%) et réanimation (20,2%). La répartition des participants selon leur qualification professionnelle était comme suit: infirmiers (tout grade et toutes spécialisations confondus) 51,3%; médecins (tout grade et toutes spécialisations confondus) 19,2% et les aides-soignants (AS), 20,2 %. La durée d'ancienneté dans l'unité de soins a été également mentionnée. Elle était inférieure à cinq ans pour 39,4% et supérieur à 5 ans pour 60,6% des enquêtés. A propos de la fréquence de travail, la majorité des enquêtés occupait un poste de travail à temps partiel (65,4%).

**Fréquence et caractéristiques du burnout:** l'échelle MBI utilisé dans notre étude révèle au moment de l'enquête 65 (63%) soignants en burnout au sein des unités de soins étudiées avec 32% de niveau faible, 48% de niveau modéré et 20% de niveau sévère. Parmi les sujets atteints de burnout, 9 (14%) étaient en EE, 5 (8%) avec une DP et 7(11%) en AP Faible. La répartition du nombre de cas de burnout en fonction des catégories professionnelles était comme suit: 12 (60 %) de médecin; 40 (63,5 %) des infirmiers et 13 (61,9 %) des aides-soignants.

**Facteurs associés au burnout:** nous n'avons pas noté d'association entre le burnout et le sexe, l'âge, la qualification professionnelle, l'ancienneté, et le rythme de travail. Une association significative positive a été trouvée entre le burnout et les caractéristiques sociodémographiques suivantes: le pavillon d'exercice (p=0,016) et la situation familiale (p = 0,013) (Tableau 1).

**Tableau 1:** Caractéristiques sociodémographiques des soignants en BO versus non BO

Caratéristiques Socio-démographiques	Non-Burnout N et %	Burnout N et %	Khi-deux
**Sexe**			NS
Masculin	16(36,4)	28(63,6)	
Féminin	23(38,3)	37(61,7)	
**Tranche d'âge**			NS
20-29ans	16(48,5)	17(51,5)	
30-39 ans	10(41,7)	14(58,3)	
40-49 ans	4(16,0)	21(84,0)	
50 ans et plus	9(40,9)	13(59,1)	
**Qualification**			NS
Médecin	8(40,0)	12(60,0)	
Infirmier(e)	23(36,5)	40(63,5)	
Aide soignant(e)	8(38,1)	13(61,9)	
**Pavillon**			P=0,016
urgences médicales	11(52,4)	10(47,6)	
urgences chirurgicales	6(37,5)	10(62,5)	
maternité	14(30,4)	32(69,6)	
réanimation	8(38,1)	13(61,9)	
**Ancienneté groupé**			NS
moins de 5 ans	19(46,3)	22(53,7)	
5 ans et plus	20(31,7)	43(68,3)	
**Statut de travail**			NS
permanent à temps plein	12(46,2)	14(53,8)	
permanent à temps partiel (service de quart)	25(36,8)	43(63,2)	
temporaire ou remplaçante	2(20,0)	8(80.0)	
**Situation familiale**			P=0,013
Avec conjoint et avec enfant(s) à charge	21(32,3)	44(67,7)	
Avec conjoint et sans enfant à charge	4(80,0)	1(20,0)	
Sans conjoint et avec enfant(s) à charge	5(45,5)	6(54,5)	
Sans conjoint et sans enfant à charge	9(39,1)	14(60,9)	

**Échelle de Siegrist:** les soignants en burnout étaient plus nombreux à avoir un ratio efforts/récompenses déséquilibré: 46,2% des soignants en burnout contre 43,6% des soignants en non burnout. Ce résultat est significativement différent. P=0,031; pas d'association avec le ration P= 0,126 (Tableau 2).

**Tableau 2:** Comparaison des réponses à l’échelle de siegrist entre soignants en burnout et en non-burnout

Valeur du ratio P=0,031	ratio inferieur a 1	22 (38,6)	35 (61,4)
	ratio supérieur ou égal à 1	17 (36,2)	30 (63,8)
Statut de surinvestissement P= 0,126	inférieur ou égal 19	19 (35,2)	35 (64,8)
	Supérieur à 19	20 (40,0)	30 (60,0)

**Caractéristiques socio professionnelles des soignants en BO versus non BO:** nous n'avons pas noté d'association entre le burnout et la communication avec les collègues, la qualité de la relation avec la l'administration, la conscience dans le travail. La prévalence du burnout était significativement associée à caractéristiques socioprofessionnelles ci-après dans le Tableau 3.

**Tableau 3:** Récapitule les données sociodémographiques et professionnelles en relation avec le burnout

Régression logistique univarié			
Variables indépendantes	Odds Ratio	IC de 95%	p-value
**Pavillon (urgences médicales réf)**			
Urgences chirurgicales	2,25	[0,12; 4,48]	0,121
Maternité	3,97	[0,16;13,61]	0,28
Réanimation	3,93	[1,16; 13,24]	0,027*
**Situation familial (Avec conjoint et avec enfant(s) à charge réf)**			
Avec conjoint et sans enfant à charge	3,79	[0,37; 30,56]	0,767
Sans conjoint et avec enfant(s) à charge	3,27	[0,33; 34,70]	0,658
Sans conjoint et sans enfant à charge	2,56	[1,22; 5,39]	0,049*
**Ratio efforts / Récompense (ratio inferieur a 1 réf)**			
Ratio supérieur ou égal à 1	2,31	[1,10; 4,84]	0,026*
**Statut de surinvestissement (inférieur ou égal 19 réf)**			
Supérieur à 19	3,06	[0,88; 10,6]	0,098
**Menacé verbal ou physique (Non réf)**			
Oui et cela ne m'a pas du tout perturbé(e)	2,60	[0,90; 7,48]	0,076
Oui et cela m'a perturbé(e)	3,75	[1,49; 9,41]	0,005*
**Opportunité changer métier (Non, certainement pas réf)**			
Non, probablement pas	1,10	[0,20; 5,94]	0,912
Oui, peut être Oui, certainement	0,93	[0,17; 5,06]	0,933
J'ai déjà un projet précis	2,85	[0,41; 19,6]	0,286
**Equilibre vie professionnelle vie privée (Autant que par le passé réf)**			
Plus autant qu'avant	3,41	[1,19; 10,7]	0,038*
Plus du tout	3,40	[0,19; 34,4]	0,476
**Ancienneté groupé (moins de 5 ans réf)**			
5 ans et plus	3,27	[0,30; 34,7]	0,308
**Tranche d'âge (20-29ans réf)**			
30-39 ans	3,79	[0,37; 30,5]	0,373
40-49 ans	0,36	[0,05; 2,68]	0,318
50 ans et plus	0,66	[0,08; 5,30]	0,702
**Suspension de travail (Non réf)**			
Oui	1,61	[0,56; 4,63]	0,377
**Régularité des oublis (Rare réf)**			
Occasionnels	1,11	[0,97; 1,28]	0,127
Régulier	4,25	[1,33; 7,91]	0,002*
**Erreurs du aux conditions de travail (Rarement réf)**			
Régulièrement	2,05	[1,52; 24,0]	0,011*

## Discussion

Cette étude a révélé un taux de burnout de 63%. Ce taux dans notre population semble en rapport avec celui trouvé par Mion *et al.* 2013 en France qui était de 62,3%. Par ailleurs, il est au-dessus de la moyenne trouvée par d'autres études qui est d'environ 51,1%. Par contre, elle était inférieure à celles trouvées chez les infirmiers du service des soins palliatifs en Tunisie (70%) [10] et peut encore s'expliquer par les populations interrogées puisque notre population de soignants se compose d'anesthésistes réanimateurs, de gynécologue-obstétriciens, d'infirmiers et médecins généralistes, d'AS, etc. Si l'on compare à présent au taux de burnout retrouvé en anesthésie réanimation/soins intensifs, ce taux est de 30 à 74,3% en fonction des études [6]. Parmi les sujets atteints de burnout, 9(14%) étaient en EE, 5 (8%) avec une DP et 7(11%) en AP Faible. Si nous choisissons de comparer les taux de burnout dans chaque dimension avec les taux de burnout des médecins, des infirmiers et AS. On conclut à un accomplissement personnel des AS plus élevé que celui des médecins et des infirmiers. Ces différences s'expliqueraient pour les auteurs par le statut particulier de chacun. Les AS ont davantage d'autonomie que les médecins et un domaine de compétences bien défini. Ils auraient en ce sens un accomplissement personnel plus élevé qu'un médecin généraliste qui est confronté au cours de sa carrière à des situations plus diverses ou qu'il n'a jamais rencontré avant, remettant en questions ses compétences à chaque fois. Par ailleurs, lorsqu'une seule dimension est touchée sévèrement, nos résultats montrent que cette dimension unique est l'épuisement émotionnel chez 14% des soignants en burnout toute profession confondue. Ce résultat peut s'expliquer au regard du modèle de Cherniss (1993) par la composition de notre population: la moitié des soignants en burnout de notre étude a entre 24 et 39 ans, soit une population jeune de soignants en début de carrière. Agoub *et al*. (2007) retrouvent une association entre l'âge et une augmentation de l'EE des infirmiers ou une baisse de l'accomplissement personnel. Un soignant en début de carrière a moins de compétences, de ressources et de connaissances vis à vis des stratégies de « coping » pour faire face aux changements et évènements stressant de sa vie professionnelle [7]. Dans la même lancée, Gabbe retrouve des taux de burnout plus élevé dans les cinq premières années d'exercice. Avec l'expérience professionnelle, la dépersonnalisation diminue et l'accomplissement personnel augmente [11].

Dans cette étude, la prevalence du burnout n'est pas influencé par le genre; de meme, d'autres études montrent l'absence d'influence du genre [12]. Mais les données sont discordantes: pour Christina Maslach, les hommes seraient plus à risque que les femmes [13]. A l'inverse, quelques études [14] incriminent le sexe féminin comme étant plus à risque de burnout et suggèrent que cela serait dû à leur vulnérabilité physique et psychologique, ensuite une plus grande émotivité à l'égard des malades et enfin, la difficulté de concilier la vie professionnelle et la vie familiale. Dans notre étude, comme dans bien d'autres [15], on ne retrouve pas de lien statistique entre l'âge et le burnout contrairement à d'autres études qui ont trouvées une association. En effet, les jeunes infirmières semblent être plus touchées par ce phénomène [16] que les plus âgées. En effet selon Truchot (2009) les soignants plus âgés et donc plus matures disposeraient de plus d'aptitudes et de compétences pour faire face au BO. Nous avons vu que l'expérience professionnelle pouvait expliquer l'évolution des trois dimensions dans le processus de burnout; elle expliquerait également en partie les différences de taux de burnout entre jeunes soignants et soignants expérimentés. Notre étude n'a pourtant pas révélé une telle différence des taux de burnout entre jeunes soignants et soignants expérimentés. Cette discordance avec la littérature peut s'expliquer par nos petits effectifs mais aussi par le seuil à cinq ans que nous avons choisi en se basant sur la durée pendant laquelle le soignant apprend à s'adapter aux conditions et situations difficiles de travail [17]. Gabbe décrit dans son étude trois phases chez la carrière d'un agrégé (« espérer » « s'adapter » « déprimer ») durant approximativement 5 ans chacune [11]. Ripp évalue une augmentation du burnout chez des internes entre la première année puis la cinquième et dernière année d'internat [18]. L'étude de Teixeira chez les anesthésistes révèle des scores de burnout moins élevés avec l'augmentation des années d'expérience professionnelle [19]. Cette différence de taux de burnout en fonction de l'ancienneté professionnelle peut aussi s'expliquer par la « sélection » des praticiens qui s'effectue au cours du temps. Choisir d'être gynécologue-obstétricien, spécialiste en santé de la reproduction, anesthésiste-réanimateur, chirurgien ou sage-femme etc c'est choisir d'être confronté à l'imprévu, aux situations émotionnellement stressantes. Les soignants qui ne supportent pas ces situations, les soignants en burnout également, ont tendance à changer de poste, de travail. On peut donc penser que les médecins et infirmiers toutes spécialités confondu qui poursuivent leur carrière au sein de ces services de l'HCY ont davantage de ressources pour faire face et s'adapter aux changements de situation. On peut assimiler cette adaptation au stress à la résolution active décrite par Cherniss (1993) dans son modèle transactionnel.

Notre étude n'a pas révélé de différence des taux de burnout entre médecins et les autres soignants. Hyman lui trouve une différence entre médecins et infirmières, concordant avec les données de la littérature sur ces deux catégories de soignants [17]. Bakker en comparant les médecins généralistes et les sages-femmes trouve une différence dans l'analyse des taux de burnout pour chaque dimension avec des taux plus élevés de dépersonnalisation et d'épuisement émotionnel chez les médecins [20]. En tant que médecin, infirmier ou AS, les contraintes professionnelles, organisationnelles ne sont pas les mêmes et pourtant aucune différence n'est notée concernant leur taux de burnout. Par contre, si on analyse les dimensions touchées par le burnout chez ces soignants, une différence apparaît alors. Nous l'avons déjà justifié plus haut dans la discussion autour des dimensions touchées. La non associativité ici pourrait aussi être due au peu d'effectif dans notre étude; mais peut être aussi dû au fait que les soignants de l'HCY sont soumis toutes catégories professionnelles aux conditions de travail différentes, ils ont de difficultés similaires sur le plan inter et intra personnel, mais pas organisationnel. On peut donc émettre l'hypothèse que les contraintes organisationnelles des médecins et autres soignants agissent de façon différente sur les composantes du burnout. Certes toute ces professions se retrouvent au carrefour de la relation d'aide, du travail routinier et autres qui sont des facteurs tresseurs tel que décrite par Cherniss (1993). Cependant, une action de prévention du burnout devrait donc tenir compte de ces différences et mériterait une étude précise de ces contraintes et de leur lien avec les dimensions du burnout sur une plus grande population pour mieux adapter la prise en charge par catégorie professionnelle. En faisant une analyse des stratégies de coping utilisées, nous nous rendons compte qu'il n'y a pas non plus de différence concernant la consommation de tabac et alcool chez les soignants en burnout. Les données de la littérature associent fréquemment burnout et abus de substance, comme en témoignent les différentes études recherchant une addiction chez les soignants, que ce soit à l'alcool, au tabac ou aux drogues [14]. Mion dans son étude chez les anesthésistes retrouve une association significative entre la prise de drogues, l'alcoolo-dépendance et le burnout [6]. Ensuite ces addictions des soignants sont essentiellement décrites parmi les anesthésistes réanimateurs (du fait de l'accessibilité aux substances) or notre population de soignants est mixte, composée de généralistes et de plusieurs spécialités. D'autre part, notre population est aussi majoritairement féminine or les addictions sont davantage décrites chez les hommes [19].

L'analyse de la communication entre collègues de travail révèle que la majorité des soignants considère pouvoir échanger avec un collègue concernant la conduite à tenir pour un patient. Cette association n'est pas significative mais témoigne d'une part de l'altération de la communication des soignants en burnout avec leurs collègues. Pour Wallace, la qualité des contacts avec les collègues de travail peut contribuer à créer un climat de collaboration qui favorise l'implication, l'estime de soi [18]. Ceci s'explique dans le modèle de Cherniss (1993) par le manque de collégialité ou les relations difficiles avec ses collègues. Dans notre étude, ces résultats témoigneraient de la solidarité qui existe entre les collègues soignant a l'HCY; ou même encore pourraient être dû à la jeunesse de notre population soignante; ils ont besoin d'entre-aide pour mieux se perfectionner dans leur travail. L'agressivité des patients est significativement associée au burnout d'après les résultats de notre étude. De même, les conduites de harcèlement des patients, leur agressivité, sont associées au burnout dans la littérature. Un patient agressif va en effet utiliser davantage de ressources du soignant et conduire par là à son épuisement. Nous pouvons émettre l'hypothèse que ce résultat se justifierait par la spécificité de la population des patients qui fréquentent cet hôpital en général et ces services en particulier. Il s'agit d'un hôpital de référence et fréquenté pour des populations mieux instruites et de ce fait plus exigeantes; d'autre part il s'agit des pavillons ou les soins doivent être fait généralement dans l'urgence, ainsi, un moindre retard ou une lenteur du soignant entrainerait l'agressivité du patient ou de ses aidants familiaux. Les soignants interrogés sont jeunes, en début de carrière professionnelle pour la plupart. La dégradation de la relation avec les patients, l'agressivité des patients, vont puiser dans les ressources du soignant tout au long de son parcours professionnel et peuvent mener chez certains à un épuisement émotionnel surtout en fin de carrière comme nous l'avons vu précédemment. De plus, notre période de collecte fait suite à une période de divers scandales survenu dans ledit hôpital et ailleurs; ceci renforcerait l'agressivité des patients recherchant davantage des meilleurs soins et prise en charge. Les soignants en mi-chemin de leur carrière professionnelle de notre étude ont peut-être été eux moins exposés à ces agressions ou du moins depuis moins longtemps, ce qui expliquerait qu'ils ont encore des ressources pour y faire face et ne pas se sentir perturbés vis à vis de ces agressions.

Dans notre étude, d'après l'analyse multi-variée, les soignants qui ne sont en couple et n'ont pas d'enfant à charge sont significativement (2,56 fois) plus en burnout par rapport a ceux qui sont en couple et avec enfant à charge OR= 2,56 avec p=0,049. Les données de la littérature décrivent le rôle essentiel du soutien social: le fait d'être en couple ou entouré socialement est représenté comme un élément protecteur du burnout [6,17] dans notre étude, les soignants en burnout sont les plus représenté parmi le personnel déclarant vivre sans conjoint et sans enfant à charge; ainsi, être célibataire est un facteur de risque; ceci peut se justifier par le fait que les célibataires sont plus impliqués davantage dans leur travail; ils ont plus de temps à consacrer à leur travail que ceux vivant en famille. Nous ne retrouvons pas cette différence d'entourage avec d'autres modalités entre soignants en burnout ou non, peut être parce que nous n'avons pas recherché le statut marital des soignants mais plutôt leur entourage au sens large (social et familial). Là où nos résultats concordent encore avec la littérature, c'est quand on regarde l'équilibre entre vie privée et vie professionnelle: comparativement à ceux indemnes du burnout, beaucoup plus de soignants en burnout estiment 3,41 fois ne plus parvenir autant qu'avant, voire plus du tout, à maintenir un équilibre entre vie privée et vie professionnelle, de façon significative (p=0,038; OR= 3,41 [1, 19 ; 10, 7]) comparativement à ceux qui le font autant que par le passé. On constate ainsi que le BO est associé à un déséquilibre au niveau de la vie en général. Ce déséquilibre pourrait parfois justifier la mauvaise attitude de certains soignants face au patient; car ils trouvent en ce dernier un stresseur responsable de sa propre situation. On pourrait donc assister au mauvais accueil, aux soins impersonnels. Le ratio efforts/ récompenses est déséquilibré pour 46,2% des soignants en burnout de notre étude comparativement aux soignants non en burnout. Par ailleurs, ces soignants en burnout présentent davantage de surinvestissement que les soignants non en burnout mais ne sont pas significativement associés. Ces résultats concordent avec l'analyse de Siegrist selon laquelle l'épuisement émotionnel est associé à un déséquilibre du ratio effort/récompense. Le surinvestissement dans la mesure où il conduit le soignant à un sur engagement et donc une aggravation du déséquilibre entre des efforts encore plus importants et des récompenses perçues faibles, contribue de façon indépendante à augmenter l'épuisement émotionnel du soignant [20]. Plus loin encore, l'analyse multi-variée montre que les soignants ayant ce déséquilibre sont fortement (2,31 fois) plus à risque de développer le BO par rapport à ceux qui ont un score de Siegrist équilibré. OR= 2.31; IC [1.10 ; 4.84] p=0.026. On comprend ainsi l'impact de la charge de travail, des heures supplémentaires au travail, du bas salaire etc, une action de prévention devrait mettre un accent particulier sur les récompenses des soignants afin d'encourager à plus d'efforts.

Dans notre étude, les personnes en burnout déclarent plus d'arrêts de travail en raison de leur épuisement que les soignants non touchés par le burnout. De même, ces soignants en burnout envisagent plus un changement de travail que les soignants non en burnout. Ces éléments sont concordants avec les données de la littérature sur le sujet. Cathébras dans une enquête auprès de médecins généralistes décrit chez 4% des soignants en burnout l'intention de quitter leur poste dans l'année et chez 32,5% de le faire sans préciser de date [14]. Gabbe décrit un tiers de professeurs agrégés susceptibles de se retirer de leur poste dans les deux ans à venir par excès de stress perçu et manque de soutien chez leur position [11]. Cependant, l'analyse multi-variée ne trouve pas d'association statistiquement significative; nos résultats peuvent une fois de plus s'expliquer par la composition de notre population de jeunes soignants et ses attentes de promotion de carrière. Siegrist constate par ailleurs que certains soignants malgré un déséquilibre entre leurs efforts fournis et leurs récompenses perçues n'envisagent pas de changer de travail mais poursuivent leurs efforts dans les situations où un déséquilibre peut être accepté temporairement pour des motifs stratégiques de carrière. Les soignants poursuivent leurs efforts en vue d'une récompense ultérieure et se refusent de suspendre le travail. On peut émettre l'hypothèse que cette situation est reproduite à l'hôpital central de Yaoundé où un jeune médecin acceptera dans l'attente d'un poste permanent hospitalier futur, de poursuivre ses efforts. Un infirmier de la même façon en vue d'un titre plus honorifique tel que le major de service, fera de même.

Les soignants en burnout dans notre étude sont significativement plus nombreux à déclarer être moins complet ou faire des oublis occasionnels ou réguliers dans la prise en charge des patients. L'association entre burnout et déclaration de ces oublis réguliers présente un odds ratio de 4,25 (IC à 95% [1]) en analyse multi variée. Donc le fait d'être en BO entraine le soignant 4,25 fois à faire plus d'oublis ou incomplétude par rapport à celui qui ne l'est pas. Ces prises en charge incomplètes ou oublis dans la prise en charge d'un patient sont sans doute constitués d'éléments parfois anodins, mais on peut faire l'hypothèse que certaines conséquences pourraient être graves tels les scandales comme celles que nous avions connues au paravent quelques mois plus tôt avant notre étude. Comme dans notre étude, les soignants en burnout dans la littérature déclarent davantage faire des oublis dans la prise en charge de leur patient, d'autant plus si ces soignants sont déprimés: burnout et dépression augmentent le taux de déclaration de ces prises en charge suboptimales [7]. On peut émettre deux hypothèses sur ce lien entre burnout et prises en charge sous optimales: un soignant en burnout présente une baisse de son accomplissement propre c'est à dire de l'estimation qu'il fait lui-même de ses compétences. Autrement dit, un soignant un burnout aura tendance à s'autodéprécier donc à déclarer plus d'oublis. L'autre hypothèse pourrait être en lien avec l'accumulation d'oublis responsables de la baisse de l'accomplissement personnel entrainant peu à peu chez le soignant un épuisement professionnel. Les soignants de notre étude pensent significativement que leurs erreurs sont dues aux conditions de travail; d'après l'analyse multivariée, les conditions de travail sont associées avec OR= 2,05; IC: [1,52; 24,0] et p= 0,011. Nous pouvons dire qu'étudier l'erreur est difficile: l'essentiel des études à ce sujet se basent sur des auto questionnaires. Or on sait que la dépression et le burnout sont associés à une baisse de l'accomplissement personnel du soignant, c'est-à-dire à son auto estimation de ses compétences pratiques. Fahrenkopf dans une étude parmi des internes de pédiatrie effectue une double enquête: d'une part une analyse des taux de burnout et des scores de dépression, d'autre part, une enquête sur les erreurs ou évènements indésirables à partir d'une analyse quotidienne des dossiers par des infirmières et médecins expérimentés objectivant ces erreurs. L'analyse des résultats témoigne d'une différence significative entre internes déprimés (six fois plus d'erreurs par internes et par mois) et internes non déprimés.

## Conclusion

Le burnout est un phénomène complexe à la frontière entre la médecine, la psychologie et la sociologie. Ce syndrome, dont la définition est encore discutée, surprend par son ampleur au sein de la population des soignants et nécessite qu'on en parle. Notre étude transversale à visée descriptive et analytique a permis de trouver une population de soignants jeunes ayant les mêmes manifestations que celles trouvées dans la littérature et parmi lesquels 63% sont en burnout. L'analyse comparative des soignants en burnout et des soignants non en burnout a permis de dégager sept éléments associés de façon significative au burnout (sur la base d'une analyse multivariée): le fait de travailler au service de réanimation/soins intensifs, le statut matrimonial, le fait d’avoir un ratio effort/récompenses déséquilibré, le fait d'avoir été menacé physiquement ou verbalement et être perturbé par cela, le fait de ne plus pouvoir maintenir un équilibre entre vie privée et vie professionnelle, la fréquence des oublis: les oublis réguliers sont fortement associés et la fréquence des erreurs et ou incomplétude dans la prise en charge des patients. Ainsi, il apparaît dans notre étude que le burnout peut frapper chaque soignant, quelque soit son ancienneté professionnelle, ou sa catégorie professionnelle. L'association du burnout à la fréquence des oublis et prises en charge incomplètes et à un ratio effort/ récompenses déséquilibré est un argument médicolégal et économique pour mettre en œuvre des changements dans l'organisation du travail afin de diminuer le burnout. Une attention particulière devra être portée sur la reconnaissance du besoin de concilier vie professionnelle et vie privée. Nous faisons état d'une situation préoccupante, chez les soignants, pouvant avoir de graves conséquences sur l'offre de soins. Il semble indispensable de se pencher au plus vite sur la santé de cette catégorie socio-professionnelle partout au Cameroun.

### Etat des connaissances actuelles sur le sujet

- L'interface “vie privée - vie au travail: effets sur l'implication organisationnelle et sur le stress perçu;
- Haute prévalence du Burnout dans les unités Tunisiennes prenant en charge des patients en fin de vie;
- L'épuisement professionnel et la satisfaction à l'égard de l'équilibre entre le travail et la vie personnelle chez les médecins américains par rapport à la population américaine en général.

### Contribution de notre étude à la connaissance

- Il montre que le syndrome de l'épuisement professionnel est fréquent chez les travailleurs de la santé dans le plus grand établissement de santé au Cameroun, Yaoundé Centre Yaoundé;
- Le syndrome de l'épuisement professionnel a un lien avec la vie en dehors du lieu de travail.

## Conflits d’intérêts

Les auteurs ne déclarent aucun conflit d’intérêts.

## References

[1] Delbrouck M, Vénara P, Goulet F, Ladouceur R (2011). Comment traiter le burn-out: principes de prise en charge du syndrome d'épuisement professionnel.

[2] Poncet MC, Toullic P, Papazian L, Kentish-Barnes N, Timsit JF, Pochard F (2007). Burnout syndrome in critical care nursing staff. Am J Respir Crit Care Med.

[3] Dupuy-Walker L, Tardif M, Lessard C (2001). Le travail enseignant au quotidien. Expérience, interactions humaines et dilemmes professionnels. Bruxelles: De Boeck Université. Revue des sciences de l'éducation.

[4] Shanafelt TD, Boone S, Tan L, Dyrbye LN, Sotile W, Satele D (2012). Burnout and satisfaction with work-life balance among US physicians relative to the general US population. Archives of internal medicine.

[5] Arora M, Asha S, Chinnappa J, Ashish D (2013). Burnout in emergency medicine physicians. Emergency Medicine Australasia.

[6] Hounkpè SB (2007). Etude du stress chez les agents de santé de deux centres hospitaliers au Bénin.

[7] Amamou B, Bannour AS, Yahia MB, Nasr SB, Ali BB (2014). Haute prévalence du Burnout dans les unités Tunisiennes prenant en charge des patients en fin de vie. The Pan Afr Med J.

[8] Gabbe SG, Melville J, Mandel L, Walker E (2002). Burnout in chairs of obstetrics and gynecology: Diagnosis, treatment, and prevention: Presidential address. Am J Obstet & Gynecol.

[9] Zeter C (2004). Burnout, conditions de travail et reconversion professionnelle chez les médecins généralistes de la région Poitou-Charentes.

[1] Maslach C, Jackson SE (1986). MBI: Maslach Burnout Inventory; manual research edition.

[11] Cathébras P, Begon A, Laporte S, Bois C, Truchot D (2004). Épuisement professionnel chez les médecins généralistes. La presse médicale.

[12] Faille A (2012). Etude descriptive de la population des médecins généralistes libéraux du Nord-Pas-de-Calais et prévalence du Burn Out: réalisée à partir de l'envoi de 1000 questionnaires (Doctoral dissertation, Thèse d'exercice.

[13] Schraub S, Marx E (2004). Le point sur le syndrome d'épuisement professionnel des soignants ou burn out, en cancérologie. Bulletin du cancer.

[14] Hyman SA, Michaels DR, Berry JM, Schildcrout JS, Mercaldo ND, Weinger MB (2011). Risk of burnout in perioperative clinicians: a survey study and literature review. Anesthesiology.

[15] Ripp J, Babyatsky M, Fallar R, Bazari H, Bellini L, Kapadia C (2011). The incidence and predictors of job burnout in first-year internal medicine residents: a five-institution study. Academic Medicine.

[16] Teixeira C, Ribeiro O, Fonseca AM (2013). Burnout in intensive care units-a consideration of the possible prevalence and frequency of newrisk factors: a descriptive correlational multicentre study. BMC Anesthesiol.

[17] Bakker AB (2009). The crossover of burnout and its relation to partner health. Stress and Health: J Inter Soc Invest of Stress.

[18] Frank E, Elon L, Naimi T, Brewer R (2008). Alcohol consumption and alcohol counselling behaviour among US medical students: cohort study. BMJ.

[19] Wallace JE, Lemaire JB, Ghali WA (2009). Physician wellness: a missing quality indicator. The Lancet.

[20] Preckel D, von Kanel R, Kudielka BM, Fischer JE (2005). Over commitment to work is associated with vital exhaustion. Inter J Occu & Env't Health.

